# Curcumin as Scaffold for Drug Discovery against Neurodegenerative Diseases

**DOI:** 10.3390/biomedicines9020173

**Published:** 2021-02-09

**Authors:** Filippa Lo Cascio, Paola Marzullo, Rakez Kayed, Antonio Palumbo Piccionello

**Affiliations:** 1 Mitchell Center for Neurodegenerative Diseases, University of Texas Medical Branch, Galveston, TX 77555, USA; filocasc@utmb.edu (F.L.C.); rakayed@utmb.edu (R.K.); 2 Departments of Neurology, Neuroscience and Cell Biology, University of Texas Medical Branch, Galveston, TX 77555, USA; 3 Department of Biological, Chemical and Pharmaceutical Sciences and Technologies-STEBICEF, University of Palermo, 90128 Palermo, Italy; paola.marzullo@unipa.it

**Keywords:** curcumin, Alzheimer’s disease, amyloid, tau

## Abstract

Neurodegenerative diseases (NDs) are one of major public health problems and their impact is continuously growing. Curcumin has been proposed for the treatment of several of these pathologies, such as Alzheimer’s disease (AD) and Parkinson’s disease (PD) due to the ability of this molecule to reduce inflammation and aggregation of involved proteins. Nevertheless, the poor metabolic stability and bioavailability of curcumin reduce the possibilities of its practical use. For these reasons, many curcumin derivatives were synthetized in order to overcome some limitations. In this review will be highlighted recent results on modification of curcumin scaffold in the search of new effective therapeutic agents against NDs, with particular emphasis on AD.

## 1. Introduction

For last two centuries, natural occurring products have attracted the attention of many researchers due to their health benefits in the prevention and treatment of several diseases [1]. In 1815 Vogel isolated a yellow pigment, called curcumin, from the rhizome of Curcuma Longa, an East Indian plant [2]. Curcumin is the most abundant polyphenol and the most biologically active molecule found in the turmeric root; other minor components, known as curcuminoids, are demethoxycurcumin, bisdemethoxycurcumin, and cyclocurcumin [3]. Curcumin is one of the main elements of the Southeast Asian diet and it has been widely used for centuries as a traditional Indian and Asian medicine. After its first extraction, several studies showed that this polyphenolic molecule exhibits a broad spectrum of biological activities. Curcumin offers several health benefits, including anticancer [4], hypoglycemic activities [5], as well as the ability to be used as an analgesic, antiseptic or antimalarial [6]. In addition, curcumin has been shown to have anti-inflammatory [7], antioxidant [8,9], and antiamyloidogenic properties, which are relevant for the treatment of Alzheimer’s disease (AD), and related diseases [10,11].

Worldwide, 50 million people have dementia. Unfortunately, this number is expected to increase exponentially, affecting 152 million people by 2050 [12]. AD is the most prevalent progressive neurodegenerative disease associated with age and the most common form of dementia [13], contributing to 60–70% of cases. AD is characterized clinically by progressive loss of memory, language problems, social withdrawal, deterioration of executive functions and eventually death [14,15]. Histopathologically, as Alzheimer’s disease progresses, the brain shrinks dramatically and is characterized by cortex damage, and progressive degeneration of limbic and cortical brain structures, mainly in the temporal lobe [15]. This atrophy also affects the cortical association areas and the hippocampus, which is critical for the formation of new memories [16]. As a result of this pattern of cortical thinning, it is also possible to observe an enlargement of ventricles and a functional alteration of Wernicke’s and Broca’s areas [17]. A common characteristic of age-related neurodegenerative diseases, including AD, is the pathological accumulation of unfolded and aggregation-prone proteins in the brain, which are considered the major cause of synaptic loss and progressive neuronal death observed in these disorders [18]. The two major systems involved in proteostasis maintenance are the autophagy-lysosomal system and the ubiquitin proteasome system [19]. However, these two systems have been found to be impaired in many neurodegenerative diseases, including AD. Therefore, the failure of these systems in maintaining proteostasis may also contribute to the pathological aggregation of proteins as well as formation of insoluble and fibrillar amyloid inclusions [20].

The major neuropathological features of AD are synaptic and neuronal degeneration and the presence of amyloid plaques and neurofibrillary tangles (NFTs).

Neuritic plaques are polymorphous aggregates made up of the amyloid Aβ peptide (Aβ) aggregates. The ≈4 kDa Aβ fragment originates from the transmembrane amyloid precursor protein (APP) by concerted proteolytic cleavage of β- and γ-secretase [21]. Monomeric Aβ1-40 (Aβ40) and Aβ1-42 (Aβ42) species can aggregate to form Aβ oligomers that can further aggregate and assembly into amyloid fibrils [22]. A growing body of evidence suggest that the oligomeric/prefibrillar Aβ peptide is the neurotoxic species that trigger the amyloid cascade, leading to the damage and eventual death of neurons associated with AD [23,24,25]. On the other hand, NFTs are intracellular inclusions of hyperphosphorylated tau, a microtubule associated protein. In its native state tau is a monomeric protein [26]. Tau is a natively unfolded protein involved in microtubule stabilization and axonal transport. However, under pathological conditions, tau can undergo abnormal post-translational modifications, including phosphorylation or acetylation [27,28]. As result of these modifications, tau detaches from the microtubules causing their disassembly, cytoskeletal instability, and axonal transport perturbation [29,30]. Unbound tau can self-aggregate forming soluble tau oligomers that assemble into paired helical filaments (PHFs) [31,32,33]. The PHFs mature into fibrils that constitute the intracellular NFTs, observed in the brain of AD patients [27]. Increasing evidence suggests that synaptic dysfunction and neuronal loss precede the formation of NFTs [34,35,36,37,38,39], indicating that the smaller and prefibrillar aggregates, tau oligomers, may be responsible for the toxic effects during the early stage of AD and other tauopathies [40,41]. Therefore, tau oligomers are considered to be highly toxic and to seed tau misfolding, thus propagating the pathology seen across different neurodegenerative diseases [38,42].

Despite the many efforts made to develop new treatments and therapeutic approaches to prevent the onset of the disease and to reverse the disease process, to date, there are no effective therapeutics. Nowadays, the therapeutic strategies available are only symptomatic treatments that counterbalance neurotransmitter disturbance, thus ameliorating a few of the clinical symptoms associated with the disease. The established treatments available are acetylcholinesterase inhibitors (e.g., Donepezil or Tacrine), antagonists of glutamate NMDA receptor (e.g., Memantine), agonist of nicotinic or muscarinic receptors, antioxidants and anti-inflammatory agents [43,44].

Growing evidence demonstrates a protective effect of curcumin against Aβ plaque formation; however, the mechanism of action is not yet fully clarified. Some studies have classified curcumin as an inhibitor of Aβ aggregation, others as disaggregating and destabilizing of amyloid fibrils [45]. In addition, curcumin has been shown to hamper Aβ oligomerization but not its fibrillization [46]. Recently, curcumin has been shown to attenuate amyloid-β aggregate-associated neurotoxicity by promoting the formation of “off-pathway” nontoxic soluble oligomers and prefibrillar proteins [47].

Curcumin has also been shown to exert a neuroprotective role by inhibiting tau aggregation. Indeed, curcumin has been shown to inhibit tau oligomerization, disintegrate preformed tau oligomers, inhibit β-sheet formation, and disaggregate tau filaments [48,49]. In addition, in vitro studies have shown that curcumin prevents the aggregation of other amyloidogenic protein, including α-synuclein (α-syn), which is a presynaptic protein involved in PD. PD is a debilitating neurodegenerative disorder characterized by the gradual loss of dopaminergic neurons in the substantia nigra pars compacta and clinically characterized as movement disorder. α-syn accumulates abnormally and aggregates in the cytosol as Lewy bodies and in the neuronal processes as Lewy neurites [50]. Several studies have showed that curcumin inhibits α–syn aggregation and reduces α-syn-induced cytotoxicity [51,52].

The neuroprotective effect of curcumin is certainly due to its ability to modulate the aggregation pathways and toxicity of amyloidogenic proteins and mitigate inflammation and oxidative stress, known to be key factors in the progression of neurodegenerative disorders [53,54].

This review attempts to explore the protective role of curcumin and its related compounds in the treatment of neurodegenerative disorders as a potential modulator of pathogenic pathways associated with AD and related diseases.

## 2. Physicochemical Characteristics of Curcumin

**Scheme 1:**
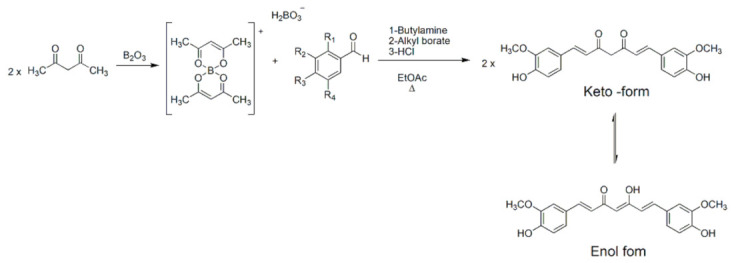
Synthesis of curcumin with Pabon’s method.

Due to the relevant biological and health benefits of curcumin, several chemists proposed a potential structure of curcumin. In 1913 Lampe et al. synthesized curcumin for the first time [55]. A general procedure for the synthesis of curcumin with a higher yield was later reported by Pabon [56]. In this reaction scheme 2,4-diketones, such as acetyl acetone, reacts with conveniently substituted aromatic aldehydes, particularly vanillin aldehyde, to synthesize curcumin. To prevent a Knoevenagel condensation due to the high acidity of the α-methylene group, the reaction is carried out in the presence of boron oxide as a complexing agent for the dienolate group. In this way, the condensation reaction involves terminal alkyl groups of di-ketone and primary or secondary amines, usually *n*-butylamine, are used to deprotonate these groups. Alkyl borates act as drying agents to remove the water formed by condensation reaction between boron complex and aromatic aldehyde. In the final step, boron complex gives the final product in acidic conditions. The reaction is refluxed, using aprotic solvent such as ethyl acetate (Scheme 1).

Several research groups follow the general method proposed by Pabon with slight modifications. For example, boron oxide has been replaced with boric acid with a lower yield [57,58]. An alternative procedure, reported by Rao et al., replaced boron oxide with borontrifluoride to obtain curcuminoid difluoroboronites that can be then hydrolyzed using aqueous methanol at pH 5.8 to get curcuminoid compounds [59]. To synthesize polyhydroxy curcuminoids, it is necessary to protect the hydroxyls groups on the starting benzaldehyde. These groups were protected as ethers and deprotected using aluminum chloride [60]. Curcumin is a low molecular mass polyphenolic compound (368.38 g/moL) with a melting point of 183 °C [61]. The IUPAC name of curcumin is 1,7-bis(4-hydroxy-3-methoxy-phenyl)-1,6-heptadiene-3,5-dione and is also known as diferuloyl methane. Curcumin is a hydrophobic molecule with a log *p* value of 3.29. It is insoluble in water and soluble in polar organic solvent, like methanol, ethanol, dimethylsulfoxide, dimethylformamide, or ethyl acetate. It is partially soluble in hexane or cyclohexane [62].

Curcumin is a symmetrical molecule composed of two aromatic rings substituted with o-methoxy phenolic groups and a β-diketone moiety as a central linker. The heptadienone linkage exhibits keto-enol tautomerism (Scheme 1) that influences physicochemical and antioxidant properties of curcumin [63,64]. Curcumin is present in its bis-keto form in acidic and neutral pH conditions (pH 3–7) due to the presence of an acid proton linked to a highly activated carbon between the two aromatic rings. Conversely, under basic conditions (pH > 8), the enol form predominately and curcumin acts as an electron donor. Indeed, the antioxidant activity of curcumin is attributed to its enolic form [65]. X-ray crystallography studies confirmed that the enol form has a lower energy as compared to the diketone tautomer and it is the exclusive form in solution [66]. Moreover, keto-enol tautomer can exist as syn and anti isomers with the syn-enol form being more stable. In the syn form the two methoxy groups are on the same side with respect to keto-enol and hydroxy groups. Thus, it is possible to identify a polar surface with either a phenolic or enol group and a nonpolar area with methoxy groups [67].

Curcumin, as well as other polyphenolic compounds, displays a strong absorption in the visible region with a maximum absorption around 410–430 nm and another band with maximum absorption at 265 nm. In the presence of nonpolar solvents, including hexane or cyclohexane, a blue-shift of the absorption spectrum is observed. Conversely, in polar solvents, such as methanol or DMSO, the peak is shifted towards the lower frequencies [65]. These observations can be justified by the shift of the keto-enol tautomerism towards the enol form in a polar solvent or towards the bis-keto form in nonpolar solvent. The enol form exhibits a larger electronic delocalization and, therefore, a red-shifted absorption peak is observed [68].

## 3. Curcumin Bioavailability: Metabolic Reactions and New Formulations

Despite the relevant biological activities of curcumin, several studies have revealed a low oral bioavailability due to its poor solubility in water, low permeability and absorption, fast metabolism, and excretion in vivo. Oral administration of curcumin in rats (500 mg/kg) showed 1% of bioavailability in rat plasma [69]. In addition, several clinical studies have revealed extremely low serum levels following oral administration [70,71]. However, curcumin bioavailability improves once it is injected intravenously in rats [72].

The gastrointestinal tract represents the first physical barrier that limits the oral absorption due to the presence of the mucus layer and the tight junction proteins [48]. In addition, following oral administration, curcumin is rapidly metabolized by both conjugation and reduction pathways in the body, resulting in the formation of several pharmacologically inactive metabolites. Indeed, O-glucoronide or O-sulphate have been the principal curcuminoid metabolites found in the plasma following oral administration in rats. Furthermore, bioreduction products such as dihydrocurcumin or tetrahydrocurcumin and their conjugates formed by alcohol dehydrogenase were identified by HPLC and mass spectrometry analyses [73]. The α,β-unsatured β-diketo moiety of curcumin can be susceptible to degradation by hydrolysis at room temperature in neutral or alkaline conditions (pH ≥ 7). Several degradation products, including ferulic acid, ferulic aldehyde, vanillin, vanillic acid, and feruloylmethane, are also found in the serum; however, the amount of conjugated metabolites is more than the amount of reduction products. Probably in biofluids the β-keto function is not free but bound to proteins and, therefore, is not hydrolysable [62,74]. Curcumin is also photoreactive and undergoes photodegradation when exposed to sunlight, forming similar products to those found following hydrolytic degradation [75,76].

Several preclinical studies have suggested curcumin as a potential therapeutic approach for AD and related diseases; however, no clinical trials have been successful. The failure of these studies may be due to curcumin low brain bioavailability after oral administration and fast metabolism [77,78]. Consequently, alternative formulations and new drug delivery systems, including liposomes and nano-based approaches, have been developed to increase curcumin brain delivery. Most of the new delivery systems proposed are characterized by the presence of a central hydrophobic pocket in which curcumin binds through hydrophobic interactions. These macromolecular systems preserve curcumin from degradation and enhance its absorption as well as its distribution [75]. Furthermore, liposomes modified with penetrating agents, including curcumin as well as other amyloids-targeting ligands, have been shown to facilitate the passage of the compound through the blood–brain barrier (BBB) and have been considered as suitable vehicle for the delivery of therapeutics in the central nervous system [79]. Recently, Giacomelli et al. developed a novel and advanced curcumin delivery system based on nanoparticles named lipid-core nano-capsules. As result of their studies, they found that curcumin loaded nano-capsules display a significantly higher neuroprotective effect against Aβ toxicity in a mouse model of AD as compared to free curcumin [80]. In addition, Yang et al. proposed a novel curcumin-loaded nanoparticle system made of chitosan and bovine serum albumin. Using this formulation, they observed an increased penetration of curcumin through the BBB and microglia activation with a subsequent increase of Aβ phagocytosis [81]. Cyclodextrins are also used as absorption enhancers in several pharmaceutical formulations. Li et al. demonstrated that α-cyclodextrin enhances intestinal absorption of curcumin via transcellular and paracellular mechanisms [82]. In recent years, the study and the analysis of several crystalline solid forms of curcumin have also been a focus of great interest due to the different physicochemical properties exhibited by these polymorphs [83,84]. In addition to these alternative formulations, Wang et al. suggested exosomes, membrane-bound extracellular vesicles, as potential delivery system of curcumin. Therefore, exosomes-derived from curcumin-treated cells were used as carrier to release selectively curcumin in the brain. This delivery system increases the percentage of curcumin crossing the BBB through receptor-mediated transcytosis. They observed a decreased phosphorylation of tau through inhibition of AKT/GSK pathway, following injection in an AD mice model [82,85].

## 4. Relationship between Structural Properties and Biological Activity of Curcumin Derivatives

The synthesis of novel curcumin derivatives represents an effective alternative to obtain curcumin analogs with a better solubility in biofluid in an effort to improve the pharmacokinetic profile of curcumin and its biological activity [48,86,87,88]. As mentioned above, curcumin acts as a neuroprotective agent blocking multiple mechanisms involved in neurodegeneration by interfering with the accumulation of misfolded aggregate proteins, including Aβ and tau, inflammation and oxidative stress. The modulation of each of these pathological pathways requires distinct structural feature of curcumin. Therefore, comprehensive structure–activity studies are extremely important to identify novel curcumin derivatives as potential therapeutic agents for neurodegenerative diseases.

In recent years, researchers have synthesized several compounds able to block Aβ fibrillogenesis. In this process, Aβ monomers aggregate to form oligomers, which then assemble to form insoluble aggregates [89]. This transformation is characterized by a structural transition from α-helix to β-sheet structure [90]. It is known that the short Aβ fragment, KLVFF (Aβ16-20) binds to full length Aβ and it is important for amyloid fibril formation [91]. A shared model hypothesizes that phenylalanine residue in the KLVFF sequence of Aβ peptides interact through Π-Π interactions during Aβ aggregation. Small molecules, such as curcumin, are able to block or break these interactions and could be valid candidates to revert amyloid formation [92].

Reinke and Gestwicki have created a library of compounds resembling curcumin structure to investigate and evaluate the effect of the three main features of curcumin on inhibition of amyloid aggregation [93]. The structural features contributing to the inhibitory potency of curcumin are the two aromatic rings, the substitution pattern of these phenyl groups, and the length and flexibility of the central linker region. To perform structural considerations and better understand which feature is critical for the inhibition of Aβ aggregation, Reinke and Gestwicki synthesized curcumin analogs by modifying only one structural feature at the time and retaining the other two. As a result of their studies, they found that compounds lacking one aromatic group are less active than curcumin, suggesting that both aromatic rings are essential to interact through hydrophobic interactions and hydrogen bonding with phenylalanine residue of Aβ monomers and inhibit amyloid formation [94]. Furthermore, hydroxyl substitution on the aromatic end group or other polar functional substituents are required for the inhibiting activity. In addition, Reinke and Gestwicki showed that both length and flexibility of the central linker region are key factors to take into consideration in the design of new Aβ aggregation inhibitors. Indeed, the inhibiting activity is negatively affected when the central linker region is too long, too short, or too flexible. The optimal length of the central linker is 8–16 Å and no more than one or two rotating sp3-hybridized carbons are required for an ideal flexibility.

It has been shown that the homeostasis of metal ions is critical for maintaining normal physiological functions. Some ions such as Al, Fe, Cu, and Zn have been observed in the brain of AD patients [95]. Their imbalance in the brain is closely related to the Aβ deposition and tau accumulation, suggesting that they play a role in the degenerative process of AD. The histidine residues of Aβ peptides (H13/H14) are good coordination sites for metal ions [96]. Curcumin can interact with metal ions forming strong complexes. Indeed, the α,β-unsaturated β-diketo moiety of curcumin has shown excellent chelating properties. Many studies reported the synthesis of stable metal-curcumin complex with a stoichiometry 2:1 (ligand:metal) [62]. Curcumin-metal complexes decrease Aβ plaques as well as suppress inflammatory processes by preventing metal induction of nuclear factor kappa B, NF-kB [97,98]; however, metal chelators can disrupt the normal brain homeostasis. Zhang et al. designed a novel curcumin derivative, named CRANAD-17, as a chelating agent to attenuate Aβ crosslinking induced by Cu [99].

Some curcumin derivatives exert their neuroprotective effects by promoting phagocytosis of Aβ fibrils. For example, it was demonstrated by Fiala et al. that bisdemethoxycurcumin (BDC) enhances macrophage-activated Aβ clearance and reduces the inflammation state [100]. Recently, Gagliardi et al. showed that the treatment of BDC derivatives in a human monocytic cell line, mimicking the peripheral blood mononuclear cells of AD, revealed overexpression of genes essential for macrophage function, including mannosyl-glycoprotein 4-beta-*N*-acetylglucosaminyltransferase and vitamin D receptor. BDC also showed a protective anti-inflammatory effect through downregulation of NF-kB and β-site APP cleaving enzyme 1 (BACE1) genes [101]. BACE 1 protein and γ-secretase enzyme cleaves APP to generate Aβ peptides, with the β-cleavage being the rate-limiting step of this sequential proteolytic pathway [102]. Inhibitors of BACE1 activity are, therefore, considered as a possible therapeutic approach and many BACE1 inhibitors have been synthesized. Two critical curcumin structural features associated with BACE 1 inhibitory activity are the phenolic rings and the unsaturated alkenyl linker between the aromatic rings. Derivatives with multiple hydroxy groups have been found to be more active than compounds with nonsubstituted phenyl groups or substituted with methoxy groups or halogen. When researchers replaced the phenol group with indole or a pyrrole ring, they produced more active compounds. These data suggest that inhibitor molecules interact with BACE 1 through hydrogen bonds [103]. Reduction products are not active against BACE 1. A planar structure is required to maintain the inhibitory activity and sp2 carbons give the optimal rigidity to the molecule. The substitution of 1,3-dicarbonyl moiety of curcumin by isosteric heterocycles, isoxazole, and pyrazole, resulted in the formation of potent inhibitors of γ-secretase enzyme [104].

Up to this point of our discussion, we have analyzed the structural aspects of curcuminoids that have been found to be important for their anti-Aβ aggregation activity. Curcumin and its derivatives also exert a protective role against misfolded tau aggregates. Unlike Aβ, tau lacks in hydrophobic residues and therefore its aggregation process does not lead to the formation of Π-Π interactions. However, under particular circumstances, the small degree of hydrophobicity, compared to other proteins, is sufficient to drive tau aggregation [105]. Tau aggregation inhibitors interact with tau by electrostatic interaction and hydrogen bonding. A recent study suggested two ruthenium-curcumin-bipyridine/phenanthroline complexes as inhibitors of tau aggregation. In these complexes, the metal ion is bound to the enol group of curcumin and to the nitrogen atoms of the ancillary ligands, bipyridine or phenanthroline. Curcumin inhibits aggregation at the nucleation stage while the positive charged ruthenium complex inhibits the elongation phase, reducing longer fibrils formation [106].

Oxidative damage plays an important role in neurodegeneration, therefore, to treat neurodegenerative disorders another pharmacological approach is to develop antioxidant compounds. The antioxidant property of curcumin is due to the abstractable phenolic hydrogen [107]. This group can reduce, for example, superoxide radicals to generate less reactive phenoxyl radicals that are resonance stabilized [108]. Ferrari et al. developed curcumin analogs substituted on the central carbon of the heptadienone linker and demonstrated that these complexes exhibit good metal chelating properties. However, these compounds showed less scavenging activity because of the presence of the substituent on central linker shifts the keto-enol tautomerism towards the di-keto form with less stabilization of phenoxyl radical [109].

## 5. Curcumin Derivatives and Hybrids Molecules

Given the large number of biologically active curcumin-like molecules that have been synthesized, we can divide them into different classes accordingly to the modified part of curcumin structure. Discussion on selected compounds is reported in the following subsections.

### 5.1. Monocarbonyl Analogs of Curcumin (MACs)

**Figure 1:**
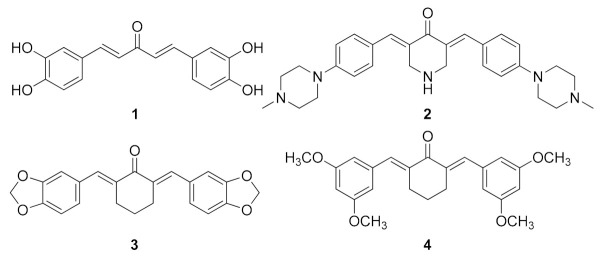
Structures of monocarbonyl analogs of curcumin (MACs).

The monocarbonyl analogs of curcumin (MACs) belong to the group of compounds with a central core modification, as shown in Figure 1. Several researchers have proposed MACs as anticancer as well as anti-inflammatory agents. These compounds have been shown to exhibit a higher anticancer potency than curcumin in many cancer cell lines [110]. Recently, monocarbonyl derivatives have also been proposed for the treatment of AD.

The removal of the keto-enol motif, susceptible to hydrolysis, enhances the stability of MACs compared to curcumin; however, the presence of the enone group is important for the anti Aβ aggregation activity [111]. MACs can be divided into two main groups: acyclic (**1**) and cyclic MAC compounds (**2**, **3** and **4**) (Figure 1). In the cyclic MACs, the carbonyl group could also be part of a 5 or 6 membered heterocyclic ring containing NR, O, S or SO_2_ groups [112]. In a general synthetic method reported by Ohori et al., MACs have been produced from aryl-aldehyde and acetone. To obtain cyclic MACs, acetone was substituted by cyclic ketone. The reaction is carried out in ethanol using sodium hydroxide and cetyltrimethylammonium bromide as catalysts [110].

Orlando et al. synthesized MAC derivatives with 5-carbon spacer between the aromatic rings. Compound **1** in Figure 1 showed a higher percentage of anti-Aβ aggregation activity compared to curcumin (IC_50_ 0.8 µM vs. IC_50_ 1.0 µM) [111]. In addition, newly synthesized monocarbonyl derivatives with a piperidone structure in the linker (**2**) are identified as potent inhibitor of Aβ fibrillation. Furthermore, substitution of a nitrogen atom within the piperidone ring with a carbon atom or *N*-methylation reduced the antiaggregation activity. The aliphatic linker gives the ideal flexibility, while the *N*-methylpiperazine groups on the two aromatic ends enhance hydrogen bond interactions with Aβ peptides. Compound **2** decreased the β-sheet structure and stabilized the α-helix content as assessed by circular dichroism spectroscopy (CD). In addition to the antiaggregating activity, compound **2** showed antioxidant and metal chelating properties [113]. MAC **3** was demonstrated to be more stable than curcumin at physiological pH. Furthermore, UV measurements of MAC **3** revealed that its spectra remained unchanged over time, while UV spectra of curcumin showed a decreased intensity and a shift towards the red. The protein hen egg white lysozyme (HEWL) was used as a model protein to study the inhibitory effect of MAC **3** on amyloid formation. MAC **3** also exhibits an optimal length of linker (8.84 Å) and it is more rigid than curcumin due to the absence of rotating sp3 carbon within the backbone. These structural features make it as an ideal inhibitor of Aβ aggregation. Fluorescence measurements using the molecular probe 8-anilinonaphatalene-sulphonate (ANS), showed decreased hydrophobic surface upon treatment with the curcumin analog MAC **3**. Docking studies demonstrated that the compound binds to the catalytic tryptophan (62 and 63) of HEWL with the carbonyl component pointing toward the hydrophilic residues of the active site [114]. The monocarbonyl-cyclohexanone derivative **4** was tested in vitro as an antioxidant agent and showed good scavenging activity for reactive oxygen species (ROS) [115]. MAC 4 exhibits antioxidant properties and protective effect against H_2_O_2_-induced cytotoxicity in PC12 cells. The compound was able to rescue the levels of glutathione (GSH) as well as the activity of superoxide dismutase and catalase. Moreover, compound **4** showed to increase mRNA expression of Nrf2, a key transcription factor of the antioxidant response [115].

### 5.2. C4-Substituted Curcumin Derivatives

**Figure 2:**
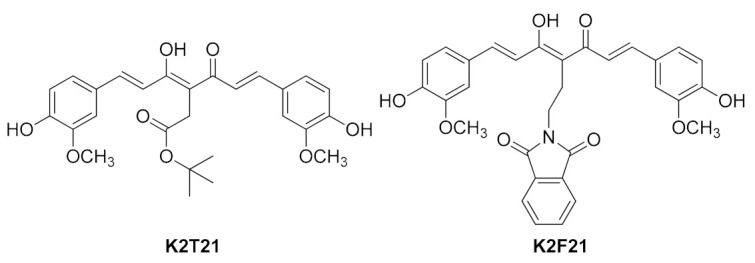
Structures of C4-substituted curcumin derivatives.

Medicinal chemists have synthesized C4-substituted curcumin derivatives by modifying the central core of curcumin with the insertion of one or two alkyl substituents (Figure 2).

The synthetic pathway follows Pabon’s route. In the first step, alkyl-substituents are inserted into an alkaline environment using acetyl-acetone and appropriate alkyl halides [116,117].

This modification as well as the synthesis of MACs, leads to the production of more stable compounds. Mono-substituted compounds have an acidic hydrogen within the linker and, therefore, retain the keto-enol tautomerism. The enolization of these derivatives is involved in the interaction with Aβ aggregates. Particularly, the anion of enol form has a reddish color while the other forms, including the neutral enol form and neutral or anion keto form, are yellow or colorless in solution. The same reddish color is observed when these molecules are incubated with Aβ aggregates, indicating an increase of the anion of the enol form when it is bound to Aβ aggregates. Notable, the interaction of the compound with Aβ monomer did not cause change in color. Therefore, this observation supports the involvement of the enol form in the binding to Aβ aggregates [118]. A decreased anti-Aβ inhibitory activity was observed for disubstituted compounds, including α,α-dimethylcurcumin. This compound lacks enol tautomer and planarity that are essential to interact with Aβ aggregates.

Ferrari et al. synthesized curcumin analogs, known as **K2T**, by introducing t-butyl ester in the C4 position [109]. **K2T** derivatives exhibit metal chelating properties towards gallium and copper ions and inhibit Aβ aggregation. The presence of alkyl substituent shifts the tautomeric equilibrium towards the di-keto tautomer and limits the radical scavenging ability. **K2T21** (Figure 2) is the best compound of this series with a good metal chelating property. In addition, the Cu(II):**K2T21** complex maintains a scavenger activity [109]. The derivative **K2F21** (Figure 2), functionalized with a phthalimide group in the α-position and vanillin-like structure on the aromatic portion, showed a higher stability, depolymerization, antioxidant and antiapoptotic activities [116]. Molecular docking simulations showed a high probability of van der Waals interactions of **K2F21** with β_2_-site of Aβ1-40 fibrils. The β_2_-binding site is located within the residues 31–40, which is a region known to be involved in modulating Aβ aggregation through the action of methionine 35 (Met35) [116].

### 5.3. Heterocyclic Derivatives

**Figure 3:**
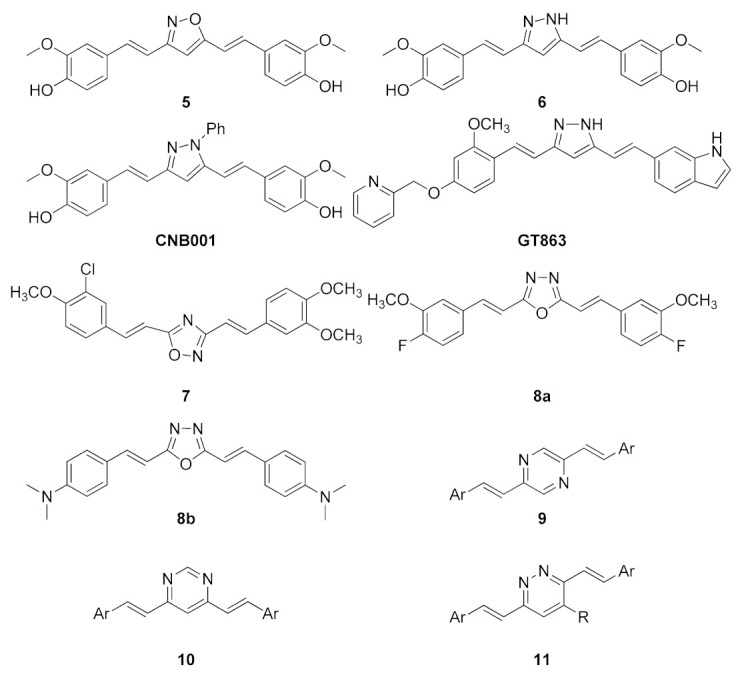
Structures of heterocyclic derivatives.

Several research groups designed and synthesized curcumin derivatives by replacing 1,3-dicarbonyl moiety with isosteric pyrazole and isoxazole rings. Isoxazole derivatives were synthesized at reflux starting from curcumin and hydroxylamine hydrochloride using pyridine and ethanol as solvents. Curcumin was converted into pyrazole and *N*-substituted pyrazole by reaction with corresponding hydrazines (NH_2_NH_2_ or RNHNH_2_), using reaction conditions reported by Narlawar et al. Particularly, from their series, compound **5** and **6** (Figure 3) are the ones exhibiting the most interesting biological effects.

Both compounds interact with Aβ42 aggregates and can be used as an imaging agent for diagnostic purposes. In addition, compound **6**, at lower concentrations, demonstrated the ability to depolymerize tau aggregates, inhibit tau aggregation, and produce γ-secretase activity [104]. **CNB001** (Figure 3) is a pyrazole analog of curcumin that has been extensively studied because of its potential as reliable therapeutic candidate for the treatment of AD. **CNB001** was shown to improve memory and long-term potentiation and mitigate motor impairments in rats [119,120]. In addition, the compound showed anti-inflammatory activity through inhibition of proinflammatory mediators in LPS-induced microglia [121]. Another compound with curcumin-pyrazole structure is **GT863** (also named PE859, Figure 3). Evaluation of its anti-Aβ and tau aggregation activity revealed that substitution of the aromatic ring with a bicyclic system along with the protection of the phenolic group from metabolic reaction, may increase the inhibitory activity [122]. It was shown that **GT863** reduces the production of Aβ through alteration of nicastrin maturation, an essential glycoprotein component of the γ-secretase complex [123].

Heterocyclic derivatives of curcumin also include compounds with an oxadiazole ring instead of a β-diketone group. Our research group has synthesized a series of curcumin-like compounds with 1,2,4-oxadiazole or 1,3,4-oxadiazole motif. Compounds **7** and **8a** were tested as Aβ aggregation inhibitors using different biophysical techniques and both showed to affect the aggregation pattern of Aβ [124]. Induced Fit Docking (IFD) observations revealed that both compounds bind to Aβ in a saddle between Met35 and Val39 via hydrophobic interactions. In the presence of compound **7** was observed a perturbation of β-sheet content, while it was partially preserved in the presence of compound **8a**. The IFD results suggest that compound **8a** can interfere with Aβ aggregation by hampering the packing of oligomers along the fibril major axis. On the other hand, due to steric hindrance, compound **7** interferes with the formation of β-sheet structure, resulting in the formation of toxic off-pathway structures. Indeed, compound **7** showed to trigger less Aβ aggregation and enhance Aβ 1-40 toxicity, probably due to the higher presence of toxic oligomers in the medium [124]. Other newly synthesized curcumin derivatives demonstrated modulation of the aggregation pathway of preformed tau oligomers. Particularly, compound **8b** was found to convert toxic tau oligomers into more nontoxic tau aggregates and mitigated tau oligomer-associated toxicity in the human neuroblastoma cell line, SH-SY5Y, and primary neuronal cultures [87].

Novel pyrimidine **9**, pyrazine **10** and pyridazine **11** curcumin derivatives, with general structure reported in Figure 1, were efficiently synthesized and tested in vitro and in vivo as potential Aβ and tau imaging probes for the diagnosis of AD. These heterocyclic derivatives showed an excellent capacity to label Aβ as well as tau aggregates [125].

### 5.4. Tetrahydrocurcumins (THCs)

**Figure 4:**
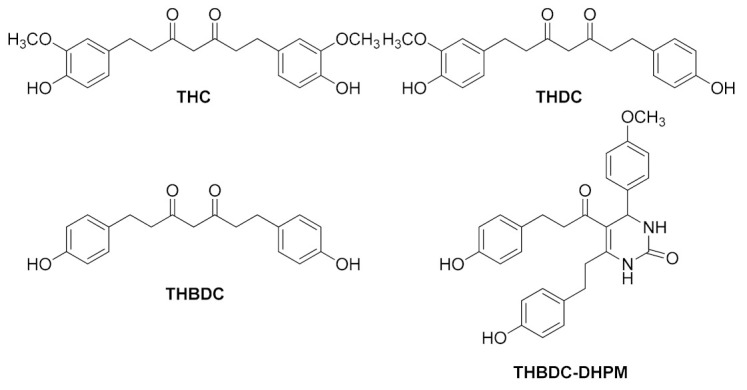
Structures of tetrahydrocurcumins (THCs) derivatives.

Hydrogenated curcuminoids attracted researcher’s attention for their biological properties (Figure 4). These compounds lack the diketone bridge necessary to bind Aβ fibrils and to exert antioxidant activity.

Tetrahydrocurcumin (**THC**) is a more stable metabolite of curcumin. Phenolic rings and methoxy groups, but not double bonds of curcumin, mediate the anti-inflammatory effect. **THC** has been shown to reduce neuroinflammation through reduction of IL1β in the brain and inhibition of LPS inducing the release of iNOS; however, it does not display any amyloidogenic inhibitor activity [126]. Tetrahydrodemethoxycurcumin (**THDMC**) and tetrahydrobisdemethoxycurcumin (**THBDMC**) are reported as inhibitors of acetyl cholinesterase activity (AChE). Particularly, the absence of the double bond and the methoxy group increases their inhibitory activity. In addition, **THCs** were conjugated with a dihydropyrimidinone, a known AChE inhibitor, to create a series of **THCs-DHPM** compounds. Compound **THBDC-DHPM** produced by **THBDMC** with methoxy group on phenyl group exhibits the lowest value of IC_50_ against the enzymatic activity [127].

### 5.5. Curcumin-Like (CL) Compounds

**Figure 5:**
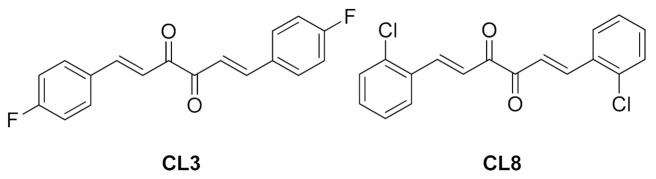
Structures of curcumin-like (CL) compounds.

To overcome the instability issues due to the β-di keto moiety, our research group synthesized a library of curcumin-like compounds without the α-carbon within the linker (Figure 5). In the synthetic method adopted, the diacetyl reacted in a double aldol condensation with the appropriate aromatic aldehyde. The resulting compounds **CL3** and **CL8** were found to modulate the aggregation state of recombinant toxic tau oligomers and disease-relevant tau oligomers [87,128]. Notably, **CL3** affected both the size and the surface hydrophobicity of brain-derived tau oligomers reducing their associated neurotoxicity [128].

### 5.6. Aromatic Ring Substitution: Methoxy and Hydroxy Groups

Aromatic rings of curcumin and curcumin-like compounds interact with Aβ aggregates by hydrophobic and hydrogen bonds. Therefore, chemical structure of curcumin containing two aromatic rings is optimal for inhibition of amyloidogenesis. Reinke and Gestwicki synthesized a series of curcumin analogs without an aromatic ring and evaluated their inhibitor activity on Aβ aggregation. These compounds did not show any effect on Aβ aggregation, even at higher concentrations (500 µM). Conversely, curcumin inhibits Aβ aggregation with an IC_50_ value of 10 µM [93].

In the same study, they also evaluated the influence of aromatic substituent on the neuroprotective activity of curcumin and its derivatives. They showed that substituents capable of taking part in hydrogen bonding are essential for the activity.

**Figure 6:**
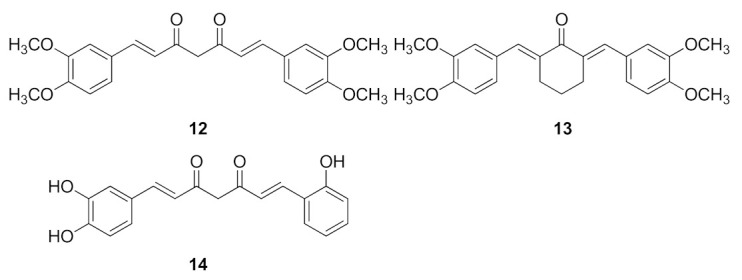
Structures of compounds with methoxy/hydroxy substituted aromatic rings.

Several structural–activity studies were performed to evaluate the effects of the position and number of methoxy and hydroxy group on the biological activity of the resulting derivatives (Figure 6).

Compounds with an anti-Aβ aggregation activity were obtained by maintaining methoxy and hydroxy group in para- and meta- positions, while orto-substitution did not improve the activity. Compound **12** was obtained from curcumin through the substitution of the para-hydroxy group with a methoxy group. Researchers observed a decreased polarity and an increased permeability across the BBB. Moreover, this substitution preserves the conjugation reaction of -OH groups with sulphate or glucuronide groups [111]. In addition, the mono-carbonyl compound **13** methoxy substituted showed an improved inhibitory activity on Aβ aggregation [114]. Hitoshi et al. showed the importance of the catechol motif for anti-Aβ aggregation activity. Particularly, they demonstrated that the o-phenol derivative **14** increases the water solubility as compared to para- and meta- phenol. Previous studies reported that an increased dihedral angle improved water solubility. In o-phenol compound hydroxyl and β-di keto groups are close and can interact by participation of water. This interaction causes a torsion of the molecule, which increases the angle between the phenol group and central linker and, thus, increases water solubility [129]. Compound **14** is a potent inhibitor of BACE1 enzyme. Computational studies suggest that the inhibitor interacts with BACE1 in the P3 pocket. It did not interact with the aspartic acid residues of active site (Asp32 and Asp228); hydroxy groups and ketone motif are involved in hydrogen bonds with Glu230 and Glu339 [103].

### 5.7. Aromatic Ring Substitution: Halogenated and Prenylated Derivatives

**Figure 7:**
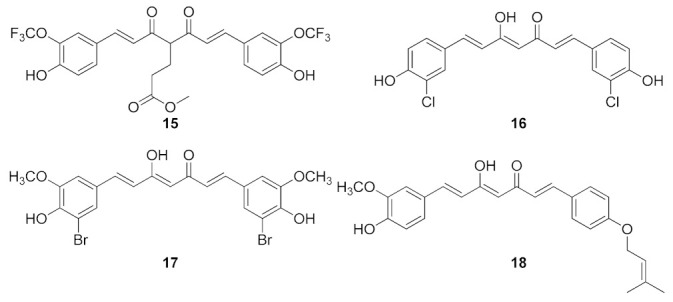
Structures of compounds with halogenated or prenylated aromatic rings.

Chemists have made several efforts to synthesize new curcumin-like compounds that can be used as diagnostic tools to detect amyloid formation in the brain. They designed the compound **15** by the introduction of tri-fluomethoxy groups on the aromatic rings (Figure 7).

This derivative passes through the BBB and reaches the brain, where it can be detected using fluorine 19 by MRI. Moreover, the trifluoromethoxy group is important for anti-Aβ aggregation activity [118]. Several studies reported other halogenated curcumin derivatives as biologically active compounds for the treatment of AD, including the chloro-substituted compound **16** [130]. In addition, the bromo-derivative **17** exhibits antioxidant properties [60]. Synthetic halogenated derivatives of curcumin have been identified as ligands for nuclear receptor of vitamin D as well as for nuclear receptor of retinoid (RXR and RAR) [131]. It was demonstrated that agonists of RXR stimulate Aβ clearance through induction of ApoE expression [132].

To increase the number of hydrogen bonding and electrostatic interactions between curcumin derivatives and Aβ peptide, a *N*-methylpiperazine group was introduced to the aromatic ring. Thus, a series of mono-carbonyl *N*-methylpiperazine substituted derivatives was synthesized and compound **2**, reported above (see Figure 1), showed the best inhibitory activity of Aβ aggregation [113]. Instead, prenylated curcumin analogs were prepared to enhance the hydrophobic contacts of curcumin with Aβ monomers. Compound **18** is the most active compound in term of antifibrillogenic and anti-inflammatory activities. It retains methoxy and hydroxy groups that are essential for H-bonds and the 4-prenyloxy group that is required to establish hydrophobic interactions with the nucleation core of Aβ peptides [133]. Indeed, the compound approaches to the self-recognition hydrophobic core, ^16^KLVFFA^21^, which is known to be a nucleation site for the pathogenic aggregation of Aβ. In particular, **18** assumes an extended geometry by interacting with six different amyloid segments in the same residues, Leu18 and Lys16. Specifically, they form hydrophobic interactions with six Leu18 residues, which contact both the phenyl ring as well as the alkyl chain. In addition, they form H-bonds with six Lys16 residues, which approach the β-keto-enol central core and the substituents on phenyl rings. When the complexity of simulated amyloid structure increases, the interactions between compound **18** and the fibril structures are governed by hydrophobic interactions [133].

### 5.8. Other Aromatic Rings

**Figure 8:**
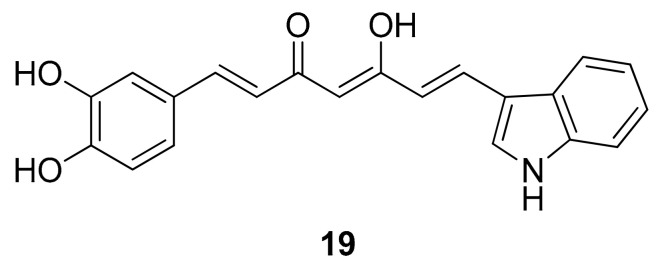
Structure of compound **19** with an indole aromatic ring.

Among lateral changes made to the structure of curcumin there is the substitution of one aromatic group with an indole moiety. The asymmetric compound **19** showed an inhibitory activity against the BACE1 enzyme and can be considered as anti-AD drug (Figure 8) [103,134]. In this compound, the substitution of phenol group with the indole motif improves the H-bonds with BACE1 enzyme.

The pyrazole curcumin derivative **GT863** (Figure 3) also presents an indole ring on the lateral part of the molecule. It inhibits both Aβ and tau aggregation. This compound is more stable than curcumin due to the presence of the pyrazole moiety and the protection of the phenolic hydroxyl group that is prone to metabolic reaction [122].

### 5.9. Hemi-Curcuminoids

**Figure 9:**
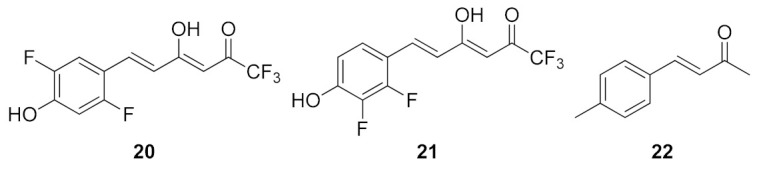
Structures of hemi-curcuminoids.

Hemi-curcuminoid β-diketones are another class of asymmetric curcumin-like compounds that can be considered the start point for the development of new neuroprotective agents (Figure 9).

These derivatives were prepared following Pabon’s method starting from substituted benzaldehydes and di-ketonic compounds. Compounds **20** and **21** display metal chelating properties and scavenger activity against ROS. The para-hydroxy group is considered a required structural feature to exert the antioxidant effect [135].

Claisen–Schmidt Aldol condensation of the aromatic aldehyde with acetone under basic conditions generated a series of hemi-curcuminoid compounds [87]. These compounds were tested as a modulator of the aggregation state of preformed tau oligomers. Compound **22** showed interaction with tau oligomers promoting the formation of larger, less hydrophobic and nontoxic tau aggregates as assessed in human SH-SY5Y neuroblastoma cells [87].

### 5.10. Calebin A Derivatives

**Figure 10:**
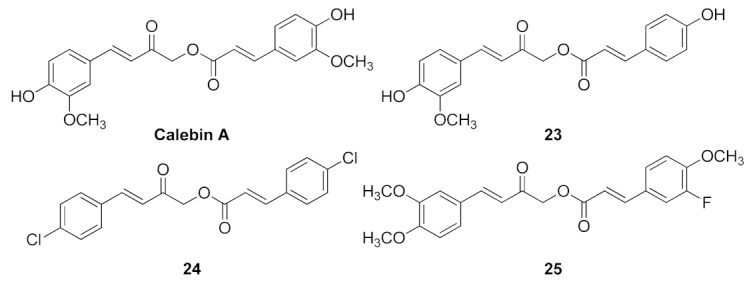
Structures of Calebin A and its derivatives.

Darrik and colleagues isolated Calebin A from Curcuma Longa and synthesized it for the first time [136]. After evaluating the protective role of Calebin A against Aβ associated toxicity in neuronal cells, several Calebin A derivatives have been synthesized (Figure 10).

Compound **23** showed that the hydroxyl group is important to protect cells from Aβ25-35 associated toxicity [137]. Calebin A derivatives are usually obtained by protecting the aromatic substituents [138]. Compounds **24** and **25** were synthesized following an alternative synthetic procedure, by substitution reaction of a cinnamic acid derivative on iodoketone in basic conditions. Both compounds were shown to protect neuronal cultures from tau oligomer-associated neurotoxicity by promoting the formation of larger nontoxic tau aggregates [87].

### 5.11. Hybrid Compounds

**Figure 11:**
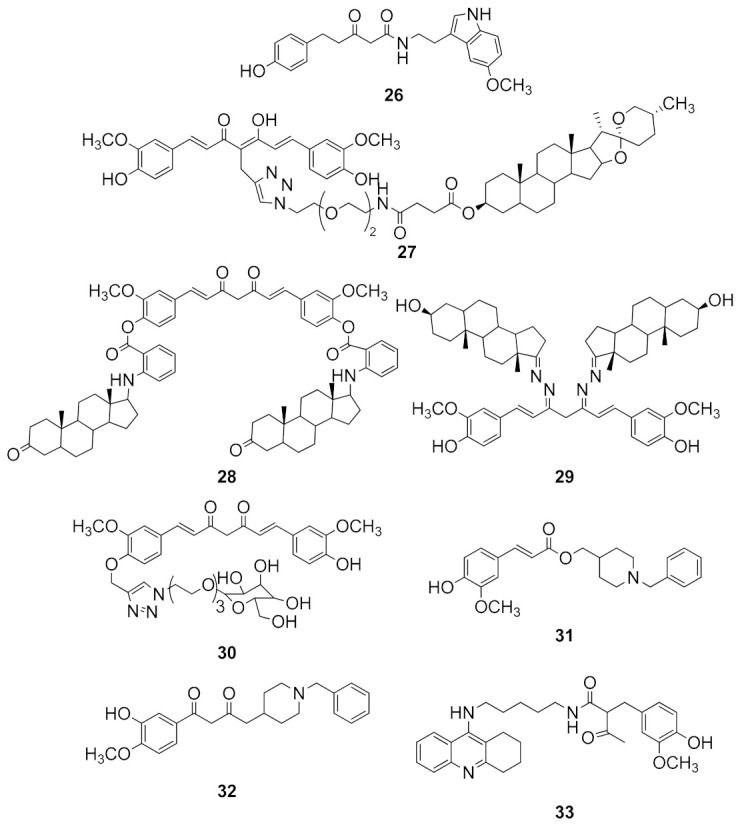
Curcumin hybrid molecules.

The bibliography about curcumin and curcumin-like compounds reports several studies on the design and biological evaluation of hybrid molecules. These compounds comprise curcumin fused with other biological active entities to enhance its biological and pharmaceutical properties (Figure 11).

Curcumin-melatonin hybrids were discovered as neuroprotective agents and have the potential to become new therapeutic strategies to treat AD. Melatonin as well as curcumin exhibit antioxidant, anti-inflammatory, and anti-Aβ aggregation activity [139]. Moreover, clinical studies have revealed that AD patients experience circadian dysfunctions due to the decreased melatonin levels in cerebrospinal fluid [140].

The presence of the methoxy group and the acetamide moiety is believed to be crucial for the neuroprotective activity of melatonin and its hybrids. Curcumin-melatonin hybrids without the p-hydroxy group of curcumin exhibited reduced neuroprotection. Contrarily, the double bound and conjugation of the β-diketone group with a phenyl ring is not necessary for the activity. The compound **26** inhibited the formation of Aβ oligomers and showed neuroprotective effects on MC65 cells [141].

Bivalent compounds were created by binding curcumin to the steroidal compound diosgenin using linkers with different lengths. The steroidal part acts as an anchor to localize curcumin into membrane lipid raft where ROS and Aβ oligomers are produced. Compound **27** with steroid moiety attached to the α-carbon of curcumin showed better neuroprotective property than those with a different position attachment. In addition, it exhibited antioxidant and anti-Aβ aggregation inhibitor activities [142]. Steroidal compounds **28** and **29** increased acetylcholine in the brain through inhibition of AChE activity and overexpression of choline acetyl transferase levels. Moreover, both compounds exert an antioxidant effect due to the phenolic groups combined to the methylene group of β-diketone group. The steroid portion can be involved in an interaction with the estrogen receptors and modulate the expression of antioxidant enzymes via intracellular pathway. Additionally, the increased levels of GSH may have a decisive role in the antioxidant effects observed. Both compounds exhibit an antiapoptotic activity as a result of the overexpression of the antiapoptotic factor Bcl2 in the brain of AD mice model [143]. Therefore, these hybrid molecules can be considered as an alternative approach to reverse the oxidative stress in neurodegeneration [143]. Additionally, bioconjugates of curcumin with demethylenated piperic acid, valine or glutamic acid display a protective effect against GSH depletion in dopaminergic neuronal cells and, therefore, may exert their antioxidant activities in the treatment of Parkinson’s disease [144]. To increase curcumin water solubility, a sugar moiety was attached to the phenol residues. The hydroxyl groups of sugar can act as β-sheet breakers through competitive hydrogen bonding with amyloids fibrils. Sugar-curcumin conjugate **30** was shown to inhibit Aβ and tau aggregation [145].

To date, acetylcholinesterase inhibitors, such as Tacrine or Donepezil, are used in the treatment of Alzheimer’s disease. Recently, new curcumin-hybrids were created by fusion of curcumin with these known drugs. Donepezil-curcumin hybrids **31** and **32** have a potent AChE inhibitor effect and both compounds showed metal chelating properties for Cu^2+^. Compound **31** also showed antioxidant activity in neuronal SH-SY5H cells. The phenyl group of the compound **31** interacts by stacking type interactions with Trp86 and the nitrogen atom binds to Tyr337 through cation-Π interaction. The carbonyl group forms a hydrogen bond with the main chain NH group of Phe295. On the other hand, compound **32** interacts with both catalytic site (CAS) and anionic site (PAS) of AChE. The two phenyl groups and the hydroxyl group interact with the active site. The hydroxyl forms an H-bond with the carbonyl group of Ser286 and Arg289, while the phenyl ring on the piperidine part formed Π-Π interactions with Trp84. The piperidine ring is located on the aromatic pocket connecting PAS and CAS sites [146,147]. Tacrine-curcumin hybrid **33** has been evaluated as an AChE inhibitor and it showed a higher potency than Tacrine [148]. Indeed, docking simulations showed that the benzene ring binds to the catalytic site of AChE, while the Tacrine motif interacts with the anionic site. The carbonyl group forms an H-bond with the Tyr121 thus stabilizing the complex. Therefore, due to the ability to inhibit both the catalytic as well as the anionic sites, compound **33** exhibited the most potent activity. Moreover, the curcumin moiety is responsible for the antioxidant properties, while the β-diketone moiety confers remarkable ion-chelating ability [148].

Biological activities of compounds discussed in this paragraph are presented in Table 1.

## 6. Conclusions

After more than two centuries from the first discovery of curcumin, its clinical applications are still under investigation. Many potential applications of this compound are envisioned but its administration and metabolic fate need to be still deeply investigated. To overcome some issues related to curcumin use as a drug candidate and address its low bioavailability, many curcumin derivatives were synthesized and tested. The field of neuroprotective compounds accounts for hundreds of different derivatives and many of them are promising drugs for the treatment of AD and related diseases. In the next years, the clinical development of these compounds will assess the real effectiveness of curcumin as a lead compound for the synthesis of novel neuroprotective drugs.

## Figures and Tables

**Table 1:** Summary of biological activities of compounds showed in Figure 1, Figure 2, Figure 3, Figure 4, Figure 5, Figure 6, Figure 7, Figure 8, Figure 9, Figure 10 and Figure 11.

Compound	Activity	Reference
MAC **1** (Figure 1)	Aβ oligomerization inhibitor	[111]
MAC **2** (Figure 1)	Aβ aggregation inhibitor Antioxidant Metal chelating	[113]
MAC **3** (Figure 1)	Aβ aggregation inhibitor Protection against Aβ oligomers toxicity Stabilization of proteins in the native state	[114]
MAC **4** (Figure 1)	Antioxidant	[115]
**K2T21** (Figure 2)	Cu (II)-chelating Antiradical	
**K2F21** (Figure 2)	Depolymerizing activity of Aβ (1–40) fibrils Antioxidant Antiapoptotic	[116]
**5** (Figure 3)	Interaction with Aβ aggregates	[104]
**6** (Figure 3)	Depolymerizing activity of tau aggregates Tau aggregation inhibitor γ-secretase activity inhibitor	[104]
**CNB001** (Figure 3)	Anti-inflammatory	[121]
**GT863** (Figure 3)	Aβ aggregation inhibitor Tau aggregation inhibitor Inhibitor of glycation of Nicastrin	[123]
**7**–**8a** (Figure 3)	Aβ aggregation modulator	[124]
**8b** (Figure 3)	Tau oligomers modulator	[87]
**9**–**10**–**11** (Figure 3)	Interaction with Aβ and tau aggregates	[125]
**THC** (Figure 4)	Anti-inflammatory	[126]
**THD**C **THBDC** **THBDC DHPM** (Figure 4)	Acetyl cholinesterase inhibitor	[127]
**CL3**–**CL8** (Figure 5)	Tau oligomers modulator	[87,128]
**12**–**13** (Figure 6)	Aβ aggregation inhibitor	[111,112,113,114]
**14** (Figure 6)	BACE1 inhibitor	[129]
**15** (Figure 7)	Aβ aggregation inhibitor Diagnostic tool to detect Aβ amyloid	[118]
**16** (Figure 7)	Aβ aggregation inhibitor	[130]
**17** (Figure 7)	Antioxidant	[60]
**18** (Figure 7)	Aβ fibrillation inhibitor	[133]
**19** (Figure 8)	BACE1 inhibitor	[103,134]
**20**–**21** (Figure 9)	Metal chelating Scavenger activity against ROS	[135]
**22** (Figure 9) **24**–**25** (Figure 10)	Modulators of tau oligomers	[87]
**23** (Figure 10)	Protection against Aβ (25–35) toxicity	[137]
**26** (Figure 11)	Aβ oligomerization inhibitor Antioxidant	[141]
**27** (Figure 11)	Aβ aggregation inhibitor Antioxidant	[142]
**28**–**29** (Figure 11)	Increase of acetyl choline levels Increase of GSH levels Antiapoptotic	[143]
**30** (Figure 11)	Aβ aggregation inhibitor Tau-aggregation inhibitor	[145]
**31**–**32**–**33** (Figure 11)	Acetyl cholinesterase inhibitor Antioxidant Metal chelating	[146,147]

## Data Availability

Not applicable.
